# Effects of alerting signals on the spatial Stroop effect: evidence for modality differences

**DOI:** 10.1007/s00426-023-01846-4

**Published:** 2023-06-30

**Authors:** Todd A. Kahan, Zachary P. Smith

**Affiliations:** Department of Psychology, Bates College, 4 Andrews Rd., Lewiston, ME 04240 USA

## Abstract

Reaction times and error rates to a target’s identity are impaired when the target is presented in a location that mismatches the response required, relative to situations where the location of the target and required response overlap (the Simon effect) and the same is true when the target's identity conveys spatial information (the spatial Stroop effect). Prior studies have found that visual versions of the spatial Stroop effect are magnified when alerting cues appear before the target and results are consistent with a dual-route framework where alerting cues boost automatic stimulus–response motor associations through the direct processing route. However, the influence of alerting signals on auditory versions of the spatial Stroop effect have not been tested and there are reasons to believe that the alerting–congruency interaction may differ across stimulus modality. In two experiments the effects of alerting cues on auditory (Experiment 1; *N* = 98) and visual (Experiment 2; *N* = 97) spatial Stroop effects are examined. Results show that alerting cues boost the spatial Stroop effect with visual stimuli but not auditory stimuli and a distributional analysis provides support for there being modality differences in the decay (or inhibition) of response-code activation. Implications for explanations of the alerting–congruence interaction are discussed.

## Effects of alerting signals on the spatial Stroop effect: evidence for modality differences

There are many instances where people await the presentation of a stimulus that will require a rapid response, and it has long been known that in these moments of anticipatory action, errors may occur. For example, in James’ (1890) chapter on attention and in Wundt’s (1874) chapter on the association of ideas, both authors note that when people anxiously await the presentation of a target stimulus that requires a speeded response, a warning signal may trigger a fast but erroneous reaction. This is not to say that warning signals are not beneficial, and data consistently show that target reaction times are faster when preceded by a warning cue (Fan et al., 2002). However, it is recognized that this benefit may come at a cost where alerting signals may also magnify the influence of distractors, which results in an alerting–congruency interaction (Callejas et al., 2005; Schneider, 2018).

For example, in the arrow version of Eriksens' flanker task (Eriksen & Eriksen, 1974; Ridderinkhof et al., 2021) participants respond to the direction of a central arrow (left or right) that is flanked on either side by distracting arrows that either point in the same (congruent), or opposite (incongruent), direction. Not surprisingly responses are faster and more accurate on congruent trials relative to incongruent trials (a congruency effect). In addition, when there is temporal uncertainty, reaction times are faster when a warning signal precedes the target’s appearance relative to when no warning signal is provided (an alerting effect). However, these two factors (congruency and alerting) often interact in a manner where congruency effects are larger when participants are alerted relative to when they had not been alerted.^1^

Whether this interaction is found, or not, may depend on the extent to which the stimuli have pre-existing directional associations. When participants respond to target stimuli with stimulus–response directional associations, then the alerting–congruency interaction consistently emerges; however, when stimuli are used that do not have directional associations congruency and alerting most often have independent effects on performance (two main effects), but do not interact.^2^ For example, the alerting–congruency interaction is robust in experiments that use arrow stimuli (see Callejas et al., 2005, among others), which have strong directional associations. Likewise, Kahan and Zhang (2019) report a robust alerting–congruency interaction in an experiment where participants categorized a centrally presented number as greater or less than 5 while ignoring flanking digits that were congruent (e.g., 33233) or incongruent (e.g., 77277) with the response required. Since participants made a left keypress response for smaller digits and a right keypress response for larger digits, and since people associate smaller numbers with the left side of space and larger numbers with the right side of space (a spatial numerical association of response codes or SNARC effect; Bächtold et al., 1998), the alerting–congruency interaction emerged here as well. Kahan and Zhang (2019) find no alerting–congruency interaction in a flanker task where participants categorized wingding characters with no directional associations. In addition, when participants respond to Stroop color words that do not have directional associations, no interaction between alerting and congruency is observed (Schneider, 2019; Soutschek et al., 2013).

The possibility that alerting signals enhance stimulus–response motor associations is further strengthened by the finding that the alerting–congruency interaction emerges in the Simon task where participants respond to a target while ignoring the location where this target appears (Böckler et al., 2011; Soutschek et al., 2013). The Simon effect is the finding that directional keypress responses, which are made to a nonspatial attribute of the target stimulus (e.g., color or shape), are faster when the target appears in a location that is congruent with the response required relative to situations where the target appears in an incongruent location (Simon & Small, 1969), and this effect is commonly explained as supporting dual-route models (e.g., De Jong et al., 1994; Kornblum et al., 1990). According to this explanation, processing takes place via a slow, intention-driven pathway that is exclusive to the target, as well as via a fast, automatic pathway that is not restricted to target features but also includes location information. When processing over the two pathways results in a mismatch in the motor responses that are activated, reaction times will be slowed relative to situations where processing via these pathways results in the activation of a single overapplying motor response. Alerting cues may exert an influence by enhancing direct route processing, which magnifies the effects seen.

Relatedly, the alerting–congruency interaction also emerges in the spatial Stroop task where participants respond to semantic aspects of the target that specify spatial directions (e.g., responding to the words left/right or left/right pointing arrows) while ignoring the location where this target appears (Fischer et al., 2010; Schneider, 2020). This procedure was first reported by Simon and Rudell (1967) in studies showing interference from the irrelevant target locations, and although this is sometimes cited as the Simon effect, this procedure is now more commonly referred to as the spatial Stroop effect (see Lu & Proctor, 1995). A critical difference between the Simon task and the spatial Stroop task, according to Kornblum’s taxonomy of stimulus–response ensembles (Kornblum, 1994), is whether the relevant dimension has overlap with the irrelevant dimension. In the spatial Stroop task the relevant and irrelevant dimensions do overlap, making this a Type 8 arrangement in Kornblum’s taxonomy (Kornblum, 1994), but this is not the case in the Simon task, which is classified as a Type 3 arrangement in Kornblum’s taxonomy. However, in a review of the literature Lu and Proctor (1995) found that both the Simon effect and spatial Stroop effect behave in much the same way, in that both are dependent on the strength of association between the irrelevant dimension and the response, and both are influenced by temporal overlap between response codes associated with the relevant and irrelevant dimensions. For this reason Proctor and Vu (2006) refer to both of these under the umbrella term Simon effects. In the current study, we focus on the spatial Stroop effect, but we also describe results from the standard Simon task where target meaning is not connected with spatial location, since these two effects may occur because of the same underlying mechanisms.

The alerting–congruency interaction has been reported in the spatial Stroop task with visually presented arrow and word stimuli (Fischer et al., 2010; Schneider, 2020), but what is not yet clear is whether the modality of the stimulus (auditory or visual), will affect the alerting–congruency interaction. To date, in all of the experiments that have examined the alerting–congruency interaction in the spatial Stroop task (Fischer et al., 2010; Schneider, 2020) only visual stimuli have been used (in fact, the same is true in experiments that have examined the alerting–congruency interaction in the Simon task^3^). However, this need not be the case. For example, in the original spatial Stroop task by Simon and Rudell (1967) participants heard the words left or right in just one ear (which randomly varied—left or right) and responded to the words while ignoring the ear. Though the spatial Stroop effect has now been reported in both the auditory and visual modality, there are two reason to believe that the alerting–congruency interaction may be larger in the visual modality relative to the auditory modality.

One possibility is that visual stimuli are more strongly linked with spatial information, while auditory stimuli are more strongly linked with temporal information. If the alerting–congruency interaction comes about because of automatic stimulus–response motor associations then the modality of the stimulus may impact the degree to which response codes associated with the irrelevant location information are activated. This possibility is supported by the sound-induced flash illusion and the ventriloquist effect. For example, when a visual and briefly flashed dot is accompanied by two auditory tones, people report seeing the dot flash twice rather than once, an effect called the sound-induced flash illusion (Shams et al., 2000; see also Hirst et al., 2020 for a review). However, when location judgments are made to sounds that are presented with conflicting visual information, location judgments are strongly influenced by vision, a finding commonly called the ventriloquist effect (Alais & Burr, 2004; Warrant et al., 1981). If vision is more strongly linked with spatial location and if the alerting–congruency interaction relies on automatic stimulus–response motor associations, then it is possible that this interaction may be stronger in a visual version of the spatial Stroop task relative to an auditory version of the task.

A second possibility is that the decay rate of task-irrelevant response codes may vary for auditory and visual stimuli and this difference in the decay rate will in turn affect the magnitude of the alerting × congruency interaction in the spatial Stroop task. This possibility is strengthened by the conclusion that both the spatial Stroop effect and the Simon effect reflect temporal overlap of response codes associated with the relevant target information and irrelevant location information (Lu & Proctor, 1995). Lu and Proctor (1995) describe several timing-based accounts that are consistent with this idea, including Hommel’s (1993) temporal overlap account which has been supported in the Simon task. For example, when activation of relevant codes is delayed by making the target stimulus less discriminable or appear gradually, there is less temporal overlap between the relevant and irrelevant response codes and the Simon effect is reduced (Hommel, 1994). In addition, if relevant codes are delayed through manipulations of stimulus eccentricity, masking, or contrast the Simon effect is also reduced (Hommel, 1993). If alerting cues extend the processing time of irrelevant response codes and if this results in greater overlap between the relevant and irrelevant response codes then the spatial Stroop effect, which may also be dependent on this overlap, should increase. The conclusion that the Simon effect is dependent on temporal overlap between relevant and irrelevant response codes is further supported by distributional analyses which show that the Simon effect decreases as reaction times (RTs) increase when visual stimuli are used (Burle et al., 2002; D’Ascenzo et al., 2018), which is expected if the irrelevant codes decay quickly, or are actively inhibited (Ridderinkhof, 2002). Importantly, this pattern of decreasing interference with longer RTs is not typically found in auditory versions of the Simon task (D’Ascenzo et al., 2018; but see Xiong & Proctor, 2016, who were able to find this pattern using stimuli that would encourage temporal overlap of the relevant and irrelevant response codes at earlier reaction time bins).

If response codes associated with auditory stimuli persist in sensory memory for a longer period than visual stimuli (Conrad & Hull, 1968) then the temporal overlap between relevant and irrelevant codes might be greater with auditory stimuli, and the spatial Stroop effect might be larger in the auditory modality as has been reported for the Simon effect (D’Ascenzo et al., 2018). Furthermore, a distributional analysis should show that alerting cues extend the spatial Stroop effect into later RT bins when visual stimuli are used but not when auditory stimuli are used, resulting in an alerting × spatial Stroop condition × bin interaction for visual stimuli.

The current experiments test these possibilities by examining the spatial Stroop effect for auditory (Experiment 1) and visual (Experiment 2) stimuli. If auditory information is less strongly associated with spatial codes than visual information, and if the effects of alerting cues are dependent on strong stimulus–response associations then a between experiment analysis should find that the spatial Stroop effect is reduced in the auditory modality. However, if temporal overlap between the relevant and irrelevant codes is critical and if auditory information remains active for an extended period relative to visual information then the opposite pattern should be found (i.e., the spatial Stroop effect should be greater in the auditory relative to visual modality). In addition, if temporal overlap is critical then a distributional analysis should show that the spatial Stroop effect is strongest at the fastest RTs for visual stimuli and alerting cues should extend this into later bins. While auditory stimuli, which persist for a longer period, should yield a large spatial Stroop effects across RT bins irrespective of the presence or absence of alerting cues.

## Experiment 1

### Methods

#### Participants

Ninety-eight students from Bates College participated in exchange for course credit in an introductory psychology or neuroscience course.^4^ We chose a sample size that was greater than other studies in this area to ensure adequate statistical power, since we anticipated that alerting may not interact with the spatial Stroop effect when auditory stimuli are used.^5^

#### Procedure

Data were collected online to reduce the spread of COVID-19 following the policies of the Institutional Review Board at Bates College. To accomplish this, all programs were created using OpenSesame software (Mathôt et al., 2012) and were generated for web delivery using PsychoPy in the back-end layer (Peirce, 2007). Programs were then hosted on a JATOS server (Lange et al., 2015). Programs ran in a web browser and participants were instructed to wear headphones or ear pods. Instructions were given at the start of the experiment with an online video which was recorded by the second author and shown in Qualtrics. Following this, participants were given a link to the experiment which started by reiterating these instructions. During all trials of the experiment the screen was blank.

Each stimulus in the experiment was presented following a randomly chosen interval which ranged from 1000 to 2500 ms. On half of the trials a warning tone (440 Hz) was presented simultaneously to both ears for 100 ms; the other trials did not include a warning tone. When this cue was presented it always preceded the onset of the target by 500 ms. Following this, participants were presented the auditory word “left” or “right” in a male voice for 250 ms. This target word was presented randomly in either the left or right ear, and since stimuli were selected in a completely randomized manner, the exact number of trials of each condition type may not have been exactly equal for each individual. Participants were required to respond to the word by pressing the “q” key with their left index finger for the word “left” and the “p” key with their right index finger for the word “right”. There was a 50% likelihood that the word and location matched (congruent trials) or mismatched (incongruent trials).

After participants completed 12 practice trials they were given visual feedback about their average accuracy and reaction time. This was followed by 8 blocks of 32 experimental trials. Average accuracy and reaction time feedback was given visually on the screen after every block of trials and participants could take self-paced breaks between blocks.

## Results

Reaction time data have a positive skew, so to reduce the influence of long reaction times which might reflect lapses in attention we analyzed geometric mean values rather than using an artificial cutoff and labeling values longer than this as spurious. To do this the log value of each reaction time on correct trials was computed and then averaged to get an average log RT for each person in each condition. The antilog of these values was then calculated, which is a geometric mean, and these geometric mean values were submitted to the ANOVAs reported. Geometric means have the benefit of keeping all values included in the analysis while reducing the influence of outliers and maintaining the same millisecond scale (see Kahan & Enns, 2010; Kahan & Zhang, 2019; Patterson & Kahan, 2022; Neill et al., 1990).

**Fig. 1:**
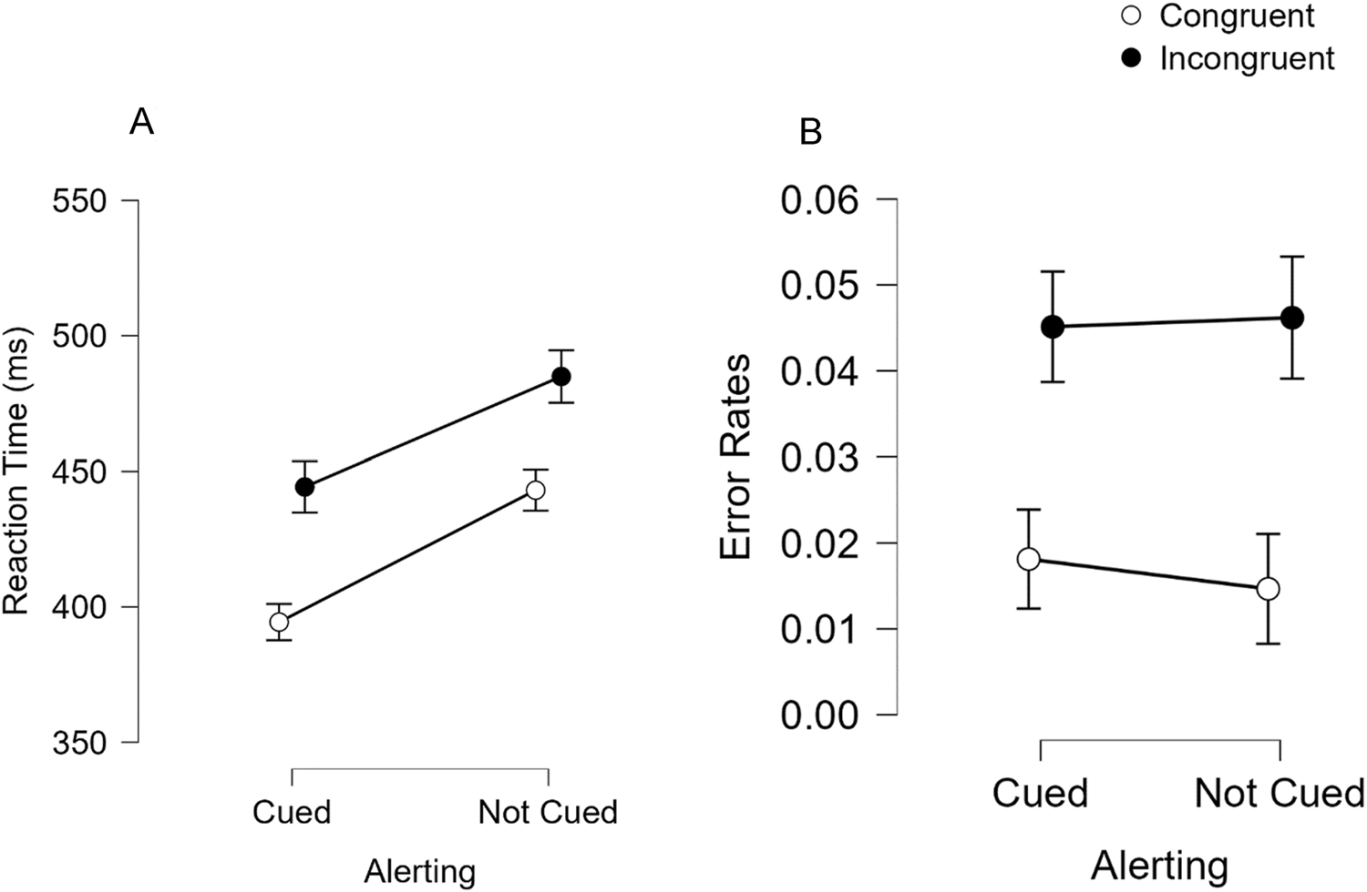
Reaction times (**A**) and error rates (**B**) to respond to respond to an auditorily presented target as a function of Alerting (cued vs. not cued) and spatial Stroop condition (congruent vs. incongruent) in Experiment 1. Error bars represent 95% confidence intervals

Reaction times from trials that were responded to correctly are shown in Fig. 1A and were analyzed in 2 (Alerting: cued vs. not cued) × 2 (spatial Stroop condition: congruent vs. incongruent) repeated measures ANOVA. There was a significant main effect of alerting, *F*(1,97) = 71.09, *p* < 0.001, which indicates that participants were faster to respond when cued (*M* = 419 ms) relative to when not cued (*M* = 464 ms). There was also a significant main effect of spatial Stroop condition, *F*(1,97) = 144.31, *p* < 0.001, which shows the spatial Stroop effect. Specifically, participants were faster when the target word and location were congruent (*M* = 419 ms) relative to when the word and location were incongruent (*M* = 465 ms). Importantly, the auditory spatial Stroop effect was not magnified by an alerting cue (50 ms when cued and 42 ms when not cued), as indicated by the lack of interaction between alerting and spatial Stroop condition, *F*(1,97) = 1.37, *p* = 0.245.

Error rates are shown in Fig. 1B and were also analyzed in 2 (Alerting: cued vs. not cued) × 2 (spatial Stroop condition: Congruent vs. Incongruent) repeated measures ANOVA. Here, the only effect that reached significance was a main effect of spatial Stroop condition, *F*(1,97) = 41.51, *p* < 0.001, which shows the spatial Stroop effect. Specifically, participants made fewer errors when the target word and ear were congruent (*M* = 0.02) relative to when the word and ear were incongruent (*M* = 0.05). The effect of alerting was not significant, *F*(1,97) = 0.26, *p* = 0.610, nor was the interaction between alerting and spatial Stroop condition (spatial Stroop effects of 0.027 when cued and 0.031 when not cued), *F*(1,97) = 0.94, *p* = 0.334.

To quantify the evidence in favor of the null hypothesis that there is no interaction, a Bayesian analysis was computed using JASP (JASP Team, 2018) for both the reaction time and error rate data. Following the recommendations of Wagenmakers et al. (2018) we present everything from each output table in Table 1. The column labeled BF_10_ indicates that compared with the null model, the model with the most support, for the reaction time data, was the model containing two main effects and no interaction. As can be seen in Table 1, adding the interaction decreases the support by a factor of 4.53 (3.32 × 10^33^/7.33 × 10^32^ = 4.53). Stated differently, the data are 4.53 times more likely under a model with two main effects compared with a model containing two main effects and an interaction between alerting and spatial Stroop condition. Similarly, for the error rate data, a model containing two main effects and no interaction is the most likely and this is 8.05 times more likely than a model with two main effects and an interaction.

**Table 1:** JASP output table for the Bayesian ANOVA of the reaction time (RT) and error rate (errors) data in Experiment 1 and 2

Expt (modality)	DV	Models	*P*(*M*)	*P*(*M*|data)	BF_M_	BF_10_	Error %
1 (Auditory)	RT	Null model (incl. subject)	0.2	2.47 × 10^–34^	9.86 × 10^–34^	1	
		Alert + SSC	0.2	0.819	18.129	3.32 × 10^+33^	4.40
		Alert + SSC + Alert * SSC	0.2	0.181	0.883	7.33 × 10^+32^	2.48
		SSC	0.2	1.61 × 10^–19^	6.46 × 10^–19^	6.55 × 10^+14^	19.03
		Alert	0.2	1.68 × 10^–20^	6.71 × 10^–20^	6.80 × 10^+13^	4.12
	Errors	Null model (incl. subject)	0.2	4.23 × 10^–15^	1.69 × 10^–14^	1	
		Alert + SSC	0.2	0.872	27.178	2.06 × 10^+14^	0.77
		Alert + SSC + Alert * SSC	0.2	0.108	0.486	2.56 × 10^+13^	3.10
		SSC	0.2	0.02	0.082	4.74 × 10^+12^	4.16
		Alert	0.2	4.85 × 10^–16^	1.94 × 10^–15^	0.115	0.93
2 (Visual)	RT	Null model incl. subject)	0.2	2.64 × 10^–22^	1.06 × 10^–21^	1	
		Alert + SSC + Alert * SSC	0.2	0.729	10.781	2.76 × 10^+21^	3.02
		Alert + SSC	0.2	0.265	1.44	1.00 × 10^+21^	2.86
		SSC	0.2	0.006	0.024	2.25 × 10^+19^	2.87
		Alert	0.2	1.50 × 10^–20^	6.02 × 10^–20^	56.962	18.43
	Errors	Null model incl. subject)	0.2	1.32 × 10^–16^	5.27 × 10^–16^	1	
		SSC	0.2	0.459	3.392	3.48 × 10^+15^	4.40
		Alert + SSC + Alert * SSC	0.2	0.436	3.091	3.31 × 10^+15^	4.81
		Alert + SSC	0.2	0.105	0.47	7.98 × 10^+14^	1.99
		Alert	0.2	3.18 × 10^–17^	1.27 × 10^–16^	0.241	1.54

The column labeled models shows the model under consideration, *P*(*M*) shows the prior model probabilities, *P*(*M*|data) shows the updated probabilities after accounting for the observed data, BF_M_ shows the extent to which the data changed the prior odds, BF_10_ shows likelihoods compared to the null model, and error % shows the numerical stability of the result based on a Markov Chain Monte Carlo (MCMC) where higher numbers indicate less stability. Models that include the factor spatial Stroop condition are depicted with the abbreviation SSC

All models include subject

**Fig. 2:**
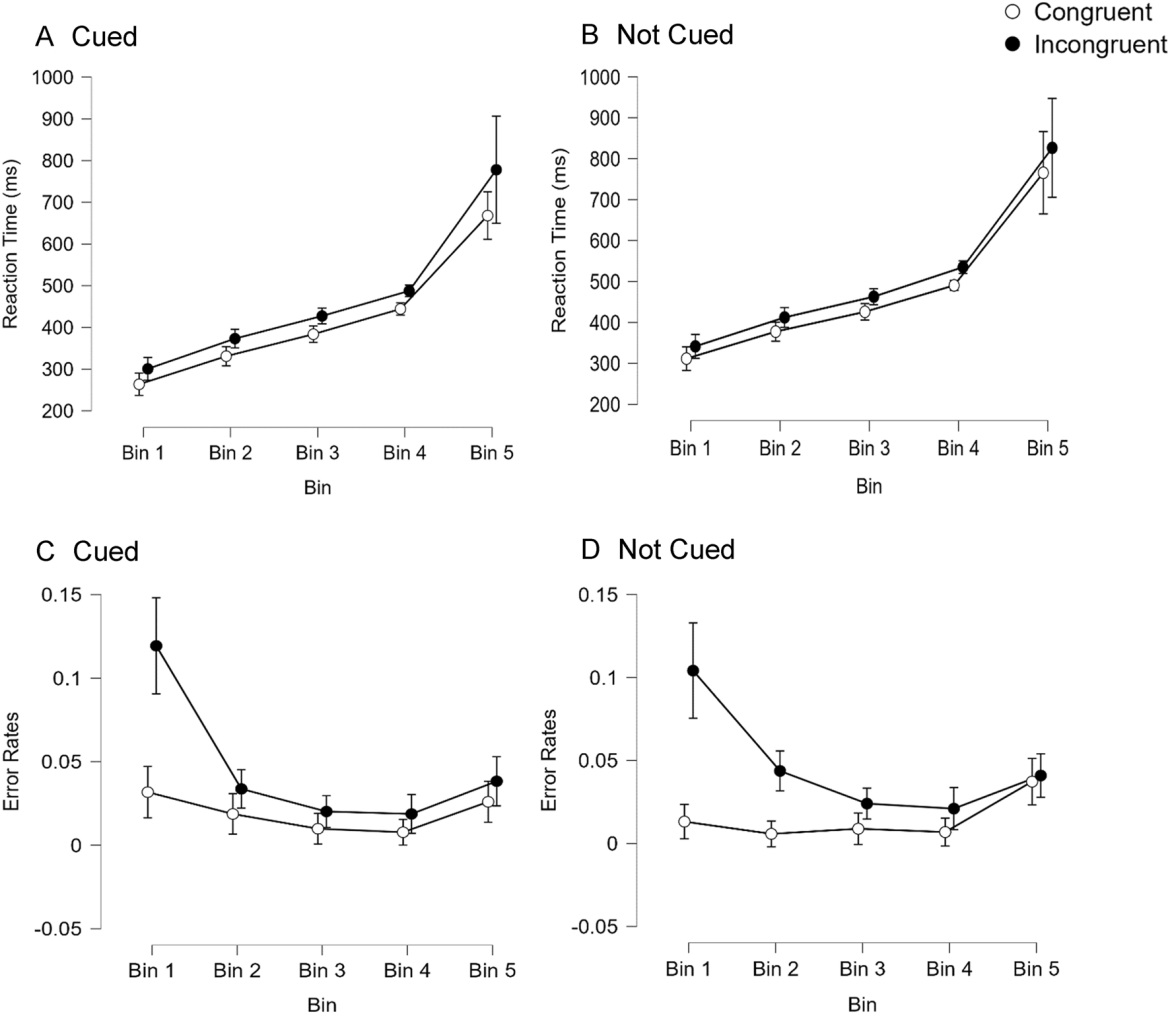
Reaction times (**A, B**) and error rates (**C, D**) to respond to an auditorily presented target as a function of Bin, spatial Stroop condition (congruent vs. incongruent), and Alerting (cued—**A, C** vs. not cued—**B, D**), in Experiment 1. Error bars represent 95% confidence intervals

A distribution analysis was also conducted to determine whether the auditory spatial Stroop effect differs for trials that are responded to more or less quickly (see Fig. 2).^6^ To do this the data were divided into 5 bins for each participant in each condition from the fastest 20% of trials (bin 1) to the slowest 20% of trials (bin 5). RT data were then analyzed in a 2 × 2 × 5 repeated measures ANOVA and the results are shown in Panel A and B of Fig. 2. This analysis yielded main effects of alerting, *F*(1,97) = 33.94, *p* < 0.001, spatial Stroop condition, *F*(1,97) = 25.80, *p* < 0.001, and bin, *F*(4,388) = 67.32, *p* < 0.001. None of the interactions approached significance (all p values > 0.25).^7^ An inspection of Fig. 2 (panels A and B) indicates that RTs were extremely variable in bin 5 and for this reason we conducted a 2 × 2 × 4 repeated measures ANOVA where the slowest bin was dropped from the analysis to examine whether its inclusion might have obscured effects observed with faster and more stable RTs. When this was done there were main effects of alerting, *F*(1,97) = 187.42, *p* < 0.001, spatial Stroop condition, *F*(1,97) = 211.02, *p* < 0.001, and bin, *F*(3,291) = 270.38, *p* < 0.001 along with an interaction between spatial Stroop condition and bin, *F*(3,291) = 3.56, *p* = 0.015. None of the other effects reached significance.^8^ This indicates that the spatial Stroop effect increased across the first 4 bins for the RT data (there was a spatial Stroop effect of 33 ms for bin 1, 39 ms for bin 2, 40 ms for bin 3, and 44 ms for bin 4). Error rates were similarly analyzed in a 2 × 2 × 5 repeated measures ANOVA and are shown in Panel C and D of Fig. 2. This analysis yielded main effects of spatial Stroop condition, *F*(1,97) = 41.28, *p* < 0.001, and bin, *F*(4,388) = 24.20, *p* < 0.001, along with two interactions. There was an interaction between alerting condition and bin, *F*(4,388) = 2.48, *p* = 0.043, which indicates that alerting cues affect performance differently for relatively fast or slow trials. The fastest trials (bin 1) were 2% less accurate when cued relative to not cued, while the slowest trials (bin 5) were 1% more accurate when cued relative to not cued. There was also an interaction between spatial Stroop condition and bin, *F*(4,388) = 2.48, *p* = 0.043, which indicates that participants made more errors on incongruent relative to congruent trials (i.e., a spatial Stroop effect) and this effect decreased as RTs slowed (there was a 9% difference in error rates for bin 1, a 2.7% difference for bin 2, a 1.3% difference for both bins 3 and 4, and 0.8% difference for bin 5). A pattern of increasing effects across bins for RTs and decreasing effects across bins for error rates has been found previously in a Simon task (Töbel et al., 2014) and this finding is discussed further in the General Discussion. None of the other main effects or interactions approached significance (all *p* values > 0.25) including the alerting × spatial Stroop condition × bin interaction, *F*(4,388) = 1.11, *p* = 0.350, which indicates that the interaction between spatial Stroop condition and bin was not influenced by the presence or absence of alerting cues.^9^

## Discussion

In this auditory version of the spatial Stroop task, which was modeled after Simon and Rudell (1967), alerting cues did not moderate the spatial Stroop effect. This result contradicts the robust interaction that has been found between alerting cues and spatial Stroop condition in visual versions of the spatial Stroop task (Fischer et al., 2010; Schneider, 2020) as well as similar studies that have found an alerting–congruency interaction in visual versions of the Simon task (Böckler et al., 2011; Soutschek et al., 2013) and is predicted if this interaction is dependent on visual presentation of stimuli. In addition, a distributional analysis indicates that the alerting × spatial Stroop condition interaction was not present at relatively fast or slow RTs (i.e., there was no three-way interaction with bin). Experiment 2 used the same experimental task (respond to the words left and right) and timing procedures but stimuli were presented visually rather than auditorily; here alerting cues are expected to magnify the spatial Stroop effect.

## Experiment 2

To verify that alerting cues magnify the spatial Stroop effect in a visual version of this task we conducted a visual analog of Experiment 1. This also serves as a conceptual replication of Schneider’s Experiment 3 which used the same basic methodology and stimulus words (left and right) but with slightly different timing parameters.

### Methods

#### Participants

Ninety-seven students from Bates College participated in exchange for course credit in an introductory psychology or neuroscience course.

#### Procedure

All aspects of the procedure were identical with Experiment 1 except here all stimuli were presented in the visual, rather than auditory, modality. Participants continued to respond to the words left or right but in Experiment 2 these words were presented visually in capital letters in 24-point Roboto Mono font. The alerting cue, when shown, were a pair of asterisks appearing simultaneously above and below fixation. Target words appeared either 160 pixels to the left of center or 160 pixels to the right of center.

The timing of events, the number of trials, the response keys used, and the feedback displays provided during breaks, were all identical with Experiment 1.

## Results

**Fig. 3:**
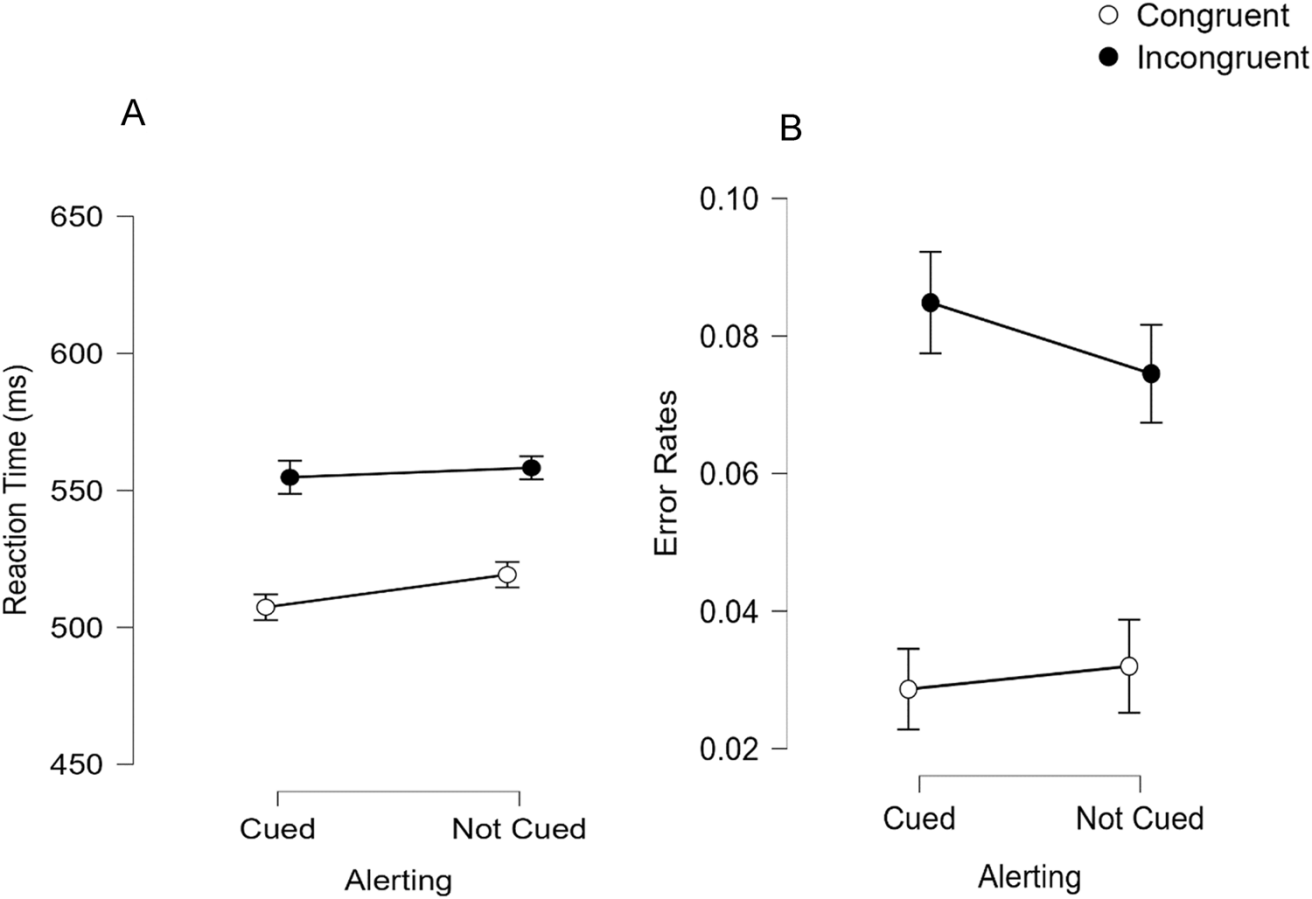
Reaction times (**A**) and error rates (**B**) to respond to a visually presented target as a function of Alerting (cued vs. not cued) and spatial Stroop condition (congruent vs. incongruent) in Experiment 2. Error bars represent 95% confidence intervals

Reaction times from trials that were responded to correctly are shown in Fig. 3A and were analyzed in 2 (Alerting: cued vs. not cued) × 2 (spatial Stroop condition: congruent vs. incongruent) repeated measures ANOVA. There was a significant main effect of alerting, *F*(1,96) = 13.69, *p* < 0.001, which indicates that participants responded more rapidly when cued in advance (*M* = 531 ms) relative to when not cued about the onset of the upcoming target (*M* = 539 ms). There was also a significant main effect of spatial Stroop condition, *F*(1,96) = 162.81, *p* < 0.001, which indicates the spatial Stroop effect was significant here. Participants responded more rapidly when the word and location on the screen were congruent (*M* = 513 ms) relative to when the word and location were incongruent (*M* = 556 ms). Importantly, there was an interaction between alerting and spatial Stroop condition, *F*(1,96) = 6.15, *p* = 0.015 (see Fig. 3A). This replicates past work examining the influence of alerting cues on the spatial Stroop effect and indicates that the spatial Stroop effect was larger following an alerting cue (48 ms) relative to when no cue was provided (39 ms).

Error rates are shown in Fig. 3B and were also analyzed in 2 (Alerting: cued vs. not cued) × 2 (spatial Stroop condition: Congruent vs. Incongruent) repeated measures ANOVA. Here, the main effect of alerting did not reach significance, *F*(1,96) = 1.77, *p* = 0.186. However, there was a significant main effect of spatial Stroop condition, *F*(1,96) = 118.69, *p* < 0.001, which indicates the spatial Stroop effect was significant in the error rate data. Participants made fewer errors when the word and location were congruent (*M* = 0.03) relative to when the word and location were incongruent (*M* = 0.08). Importantly, the interaction between alerting and spatial Stroop condition was also significant, *F*(1,96) = 5.88, *p* = 0.017 (see Fig. 3B). The spatial Stroop effect was larger following an alerting cue (5.6% difference in error rates) relative to when no cue was provided (4.3% difference in error rates).

A Bayesian analysis was also conducted on the RT and error rate data (see Table 1). For the RT data a model with two main effects and an interaction was the most likely and this was 2.76 times more likely than a model with two main effects and no interaction (2.76 × 10^21^/1.00 × 10^21^ = 2.76). This same comparison for the error rate data shows that a model with two main effects and an interaction was 4.15 times more likely than a model with two main effects and no interaction (3.31 × 10^15^/7.98 × 10^14^ = 4.15).

**Fig. 4:**
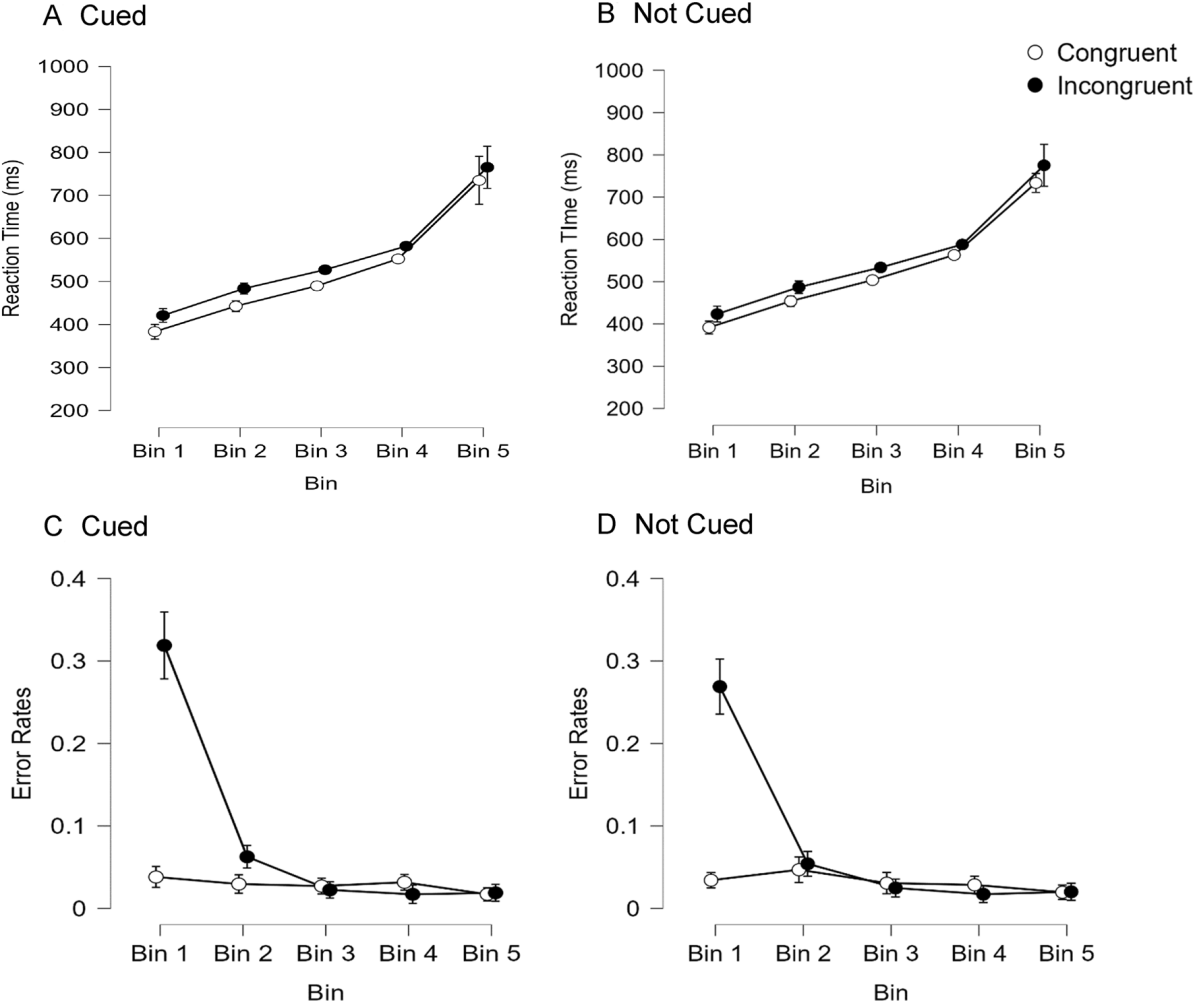
Reaction times (**A, B**) and error rates (**C, D**) to respond to a visually presented target as a function of Bin, spatial Stroop condition (congruent vs. incongruent), and Alerting (cued—**A, C** vs. not cued—**B, D**), in Experiment 2. Error bars represent 95% confidence intervals

To determine whether the visual spatial Stroop effect differs for trials that are responded to more or less quickly a distribution analysis was also conducted (see Fig. 4). To do this the data were divided into 5 bins for each participant in each condition in the same manner as Experiment 1. RT data were analyzed in a 2 × 2 × 5 repeated measures ANOVA and the results are shown in Panel A and B of Fig. 4. This analysis yielded main effects of alerting, *F*(1,95) = 4.82, *p* = 0.031, spatial Stroop condition, *F*(1,95) = 114.19, *p* < 0.001, and bin, *F*(4,380) = 138.53, *p* < 0.001. None of the other effects reached significance including the alerting × spatial Stroop condition interaction, *F*(1,95) = 0.25, *p* = 0.616.^10^ A visual inspection of Fig. 4 (Panels A and B) indicates that RTs were extremely variable in bin 5 and for this reason we conducted a 2 × 2 × 4 repeated measures ANOVA where the slowest bin was dropped from the analysis to examine whether its inclusion might have obscured effects observed with faster and more stable RTs (as had been done in Experiment 1). When this was done there was main effects of alerting, *F*(1,95) = 23.24, *p* < 0.001, spatial Stroop condition, *F*(1,95) = 201.36, *p* < 0.001, and bin, *F*(3,285) = 542.18, *p* < 0.001 along with an interaction between alerting and spatial Stroop condition, *F*(1,95) = 5.83, *p* = 0.018. This indicates that the spatial Stroop effect was larger when alerted (37 ms) relative to when not alerted (30 ms). There was also an interaction between spatial Stroop condition and bin, *F*(3,285) = 6.00, *p* < 0.001, which indicates that the spatial Stroop effect decreased with slower RTs (there was a spatial Stroop effect of 35 ms for bin 1, 37 ms for bin 2, 33 ms for bin 3, and a 27 ms for bin 4). None of the other effects reached significance.^11^ Error rates were similarly analyzed in a 2 × 2 × 5 repeated measures ANOVA and are shown in Panel C and D of Fig. 4. This analysis yielded main effects of spatial Stroop condition, *F*(1,96) = 123.36, *p* < 0.001, and bin, *F*(4,384) = 167.16, *p* < 0.001, but the main effect of alerting was not significant, *F*(1,96) = 2.00, *p* = 0.160. All of the interactions were significant. The interaction between alerting and spatial Stroop condition, *F*(1,96) = 6.08, *p* = 0.015, indicates that the spatial Stroop effect was larger when cued (5.9% more errors in the incongruent condition relative to congruent condition) relative to when not cued (4.5% more errors in the incongruent condition relative to congruent condition). The, interaction between alerting and bin, *F*(4,384) = 4.85, *p* < 0.001, indicates that the alerting cue hurt performance in bin 1 (2.6% more errors when cued), and this effect goes away at later bins (differences of 0.5%, 0.3%, 0.1%, and 0.2% for bins 2–5). The, interaction between spatial Stroop condition and bin, *F*(4,384) = 156.35, *p* < 0.001, indicates that participants made more errors on incongruent relative to congruent trials (i.e., a spatial Stroop effect) and this effect decreased as RTs slowed (there was a 25.8% difference in error rates for bin 1, a 2% difference for bin 2, a − 0.5% difference for both bin 3, a − 1.3% difference for bin 4, and a 0.2% difference for bin 5). Most importantly, the three-way interaction between alerting, spatial Stroop condition, and bin was also significant, *F*(4,384) = 2.52, *p* = 0.041. This indicates that the alerting × spatial Stroop condition interaction (where the spatial Stroop effect is larger when participants are cued relative to when not cued) decreases across bins. Specifically, the alerting–congruency interaction was significantly greater than zero for bin 1, *t*(96) = 1.99, *p* = 0.049, and bin 2, *t*(96) = 2.01, *p* = 0.047, but this was not significant for bin 3, *t*(96) = 0.14, *p* = 0.890, bin 4, *t*(96) = 0.42, *p* = 0.677, or bin 5, *t*(96) = 0.20, *p* = 0.840.

## Between-experiment analysis

In addition, we examined all of the data across experiments in a 2 (Alerting: cued vs. not cued) × 2 (spatial Stroop condition: congruent vs. incongruent) × 2 (Experiment: auditory vs. visual) mixed ANOVA to (1) determine if the overall magnitude of the spatial Stroop effect differs across modality (i.e., an Experiment × spatial Stroop condition interaction) and (2) further verify that the interaction between Alerting and spatial Stroop condition was present in the visual modality, but not the auditory modality, which should appear as a three-way interaction. We recognize, however, that the power to detect this three-way interaction across participants may be low, since the means in most of the conditions are going in the same direction, but to slightly different degrees across experiments.

The interaction between Experiment and spatial Stroop condition was not significant in the RT data, *F*(1,193) = 0.27, *p* = 0.602, but this was significant in the error rate data, *F*(1,193) = 9.82, *p* = 0.002. This indicates that while the size of the spatial Stroop effect (averaging across alerting) does not differ between the auditory (46 ms) and visual (43 ms) modalities in the RT data, for the error rate data the spatial Stroop effect was larger in the visual modality (5% more errors on incongruent relative to congruent trials) relative to the auditory modality (3% more errors on incongruent relative to congruent trials).

**Fig. 5:**
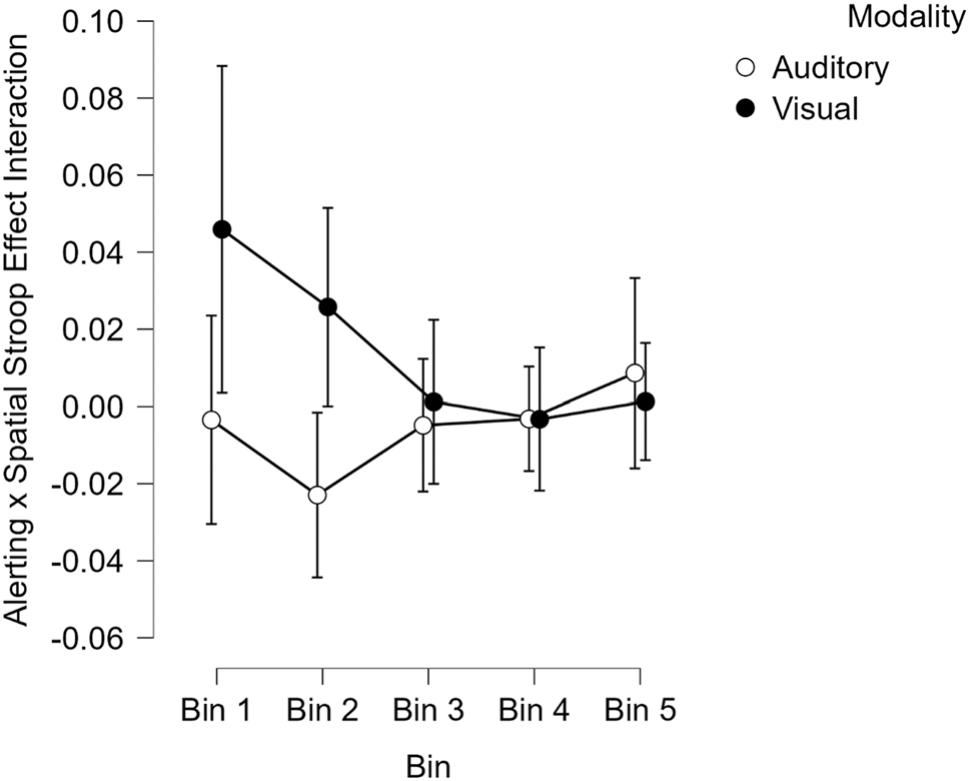
Alerting × spatial Stroop effect interaction (positive values indicate a larger spatial Stroop effect when cued relative to not cued) as a function of modality (Auditory—Experiment 1 vs. Visual—Experiment 2) and bin for error rate data. Error bars represent 95% confidence intervals

In terms of the second question of whether the alerting × spatial Stroop condition interaction differs across modality, the three-way interaction did not approach significance in the reaction time data, *F*(1,193) = 0.00, *p* = 0.962. Importantly, the three-way interaction between spatial Stroop condition, alerting, and modality did reach significance in the error rate data, *F*(1,193) = 6.20, *p* = 0.014, which provides additional evidence that the effect of alerting cues on the spatial Stroop effect depends on stimulus modality. Finally, a 2 (Alerting: cued vs. not cued) × 2 (spatial Stroop condition: congruent vs. incongruent) × 2 (Experiment: auditory vs. visual) × 5 (Bin) mixed ANOVA was conducted on both the RT and Error rate data to determine whether the alerting × congruency interaction that was found for visual stimuli (Experiment 2) but not auditory stimuli (Experiment 1) differed across response bins. The four-way interaction did not approach significance in the RT data, *F*(4,768) = 0.42, *p* = 0.792, but was significant in the error rate data, *F*(4,772) = 2.60, *p* = 0.035 (see Fig. 5). This indicates that for error rates, the alerting × spatial Stroop condition interaction occurs with visual stimuli (Experiment 2) but not auditory stimuli (Experiment 1) in bins 1 and 2, but not bins 3, 4, or 5. This can be seen in Fig. 5 where the 95% confidence intervals do not contain a value of zero in the first two bins with visually presented stimuli.

## Discussion

In a visual version of the spatial Stroop task the spatial Stroop effect was larger when participants were cued in advance relative to when they were not cued (i.e., an alerting × spatial Stroop condition interaction) and this was true in both the RT data and the error rate data. This replicates the findings of Fischer et al. (2010) and Schneider (2020). Furthermore, a distribution analysis shows that the spatial Stroop effect decreases across reaction-time bins for both RTs and error rates, and that alerting cues boost the size of the spatial Stroop effect in the error rate data in the first two bins but not later bins.

## General discussion

Across two experiments the influence of alerting signals on the spatial Stroop effect was examined. In Experiment 1, where auditory stimuli were used, alerting cues facilitated responses and a robust spatial Stroop effect was observed, yet these two factors did not interact. In addition, a Bayesian analysis provides positive evidence for the null hypothesis (Jeffreys, 1961; Raftery, 1995) that alerting signals do not magnify the auditory spatial Stroop effect. In Experiment 2, where visual stimuli were used, alerting cues did increase the visual spatial Stroop effect in a manner analogous to what has been reported elsewhere (Fischer et al., 2010; Schneider, 2020) and a distributional analysis shows that for the error rate data this boosting of the spatial Stroop effect by alerting cues is confined to the fastest RTs (see Fig. 5).

One methodological concern with these experiments, which were conducted online to reduce the spread of COVID-19, is that we did not have a way of verifying that headphones were worn in Experiment 1. We believe this was not a significant factor, though because if spatial location were not extracted in Experiment 1, then the magnitude of the spatial Stroop effect should have been significantly lower in the auditory modality than the visual modality, and this was not the case. Similarly, the number of participants who exhibited a spatial Stroop effect did not differ across modality with 91 participants showing a spatial Stroop effect in the Auditory modality and 92 participants showing this effect in the visual modality, *χ*^2^ (1) = 0.33, *p* = 0.564. Another methodological factor that was not examined here is the modality of the cue. In our experiments auditory stimuli were preceded by an auditory cue and visual stimuli were preceded by a visual cue. We note that both cue types have successfully produced alerting × congruency interactions in prior studies (e.g., Fischer et al., 2012; Kahan & Zhang, 2019; Schneider, 2019; to name just a few), so we do not believe the cue’s modality (auditory vs. visual) caused the differences seen here, but this awaits further testing. In addition, the location of our visual cues was above and below fixation (which is a methodology that has been used with success previously; Kahan & Zhang, 2019; Patterson & Kahan, 2022) rather than presenting visual cues to the left and right of fixation and cue location might impact the alerting × congruency interaction. The influence of these factors awaits further testing.

A common explanation for both the Simon effect and the spatial Stroop effect is that these effects result from activation within a dual-route framework (e.g., De Jong et al., 1994). According to this account processing takes place over two pathways; one pathway is slow, controlled, and limited to the target and the other is fast, automatic, and not restricted to target features. The controlled pathway extracts target information and transforms this into the necessary response codes, while the automatic pathway activates response codes associated with the target as well as response codes that are activated by the target’s location. When the two pathways activate the same response, performance is improved, but when processing along these pathways results in response competition, performance is worsened. If alerting cues boost automatic stimulus–response associations that are activated via the direct processing pathway, then the spatial Stroop effect ought to be enhanced by alerting cues (Fischer et al, 2010). However, this account, without modification, does not explain the modality effects seen here and in what follows we offer two possible explanations for differences across modality.

One possibility is that the alerting × spatial Stroop condition interaction is dependent on automatic stimulus–response directional associations, and auditory stimuli do not engage directional associations in the same manner as visual stimuli. The hypothesis that visual cues are more strongly linked with spatial locations than auditory cues is supported by the ventriloquist effect (Alais & Burr, 2004; Warrant et al., 1981) and the finding that the spatial Stroop effect was larger in the error rate data in Experiment 2 (visual modality) relative to Experiment 1 (auditory modality). The problem with this explanation, however, is that Experiment 1 is unlike the ventriloquist effect, since there were no conflicting visual signals to compete with auditory location information. In addition, location information must have been extracted from the auditory signals, since a robust spatial Stroop effect was found in this modality. If auditory signals did not activate location information then no spatial Stroop effect would have been found. In addition to these issues, a distributional analysis indicates that for the visual spatial Stroop effect, the alerting × spatial Stroop condition interaction in the error rate data only emerged at the fastest RTs. This pattern is not predicted by an account based solely on visual information being linked more strongly with spatial location than the auditory information.

Another possible explanation for the interaction between alerting cues and the spatial Stroop effect is that alerting cues boost activation levels and extend the processing time of target and non-target response codes by delaying the rate of decay. If this were to occur then the influence of stimulus–response associations from the irrelevant dimension may be enhanced. Furthermore, to the extent that auditory stimuli persist in sensory memory for a longer period than visual stimuli (Conrad & Hull, 1968), it is possible that alerting cues will not extend the processing time of codes associated with a slowly decaying auditory trace, but will extend the processing time of quickly fading codes that were activated by visual information. This might lead to the prediction that the spatial Stroop effect should be larger in the auditory modality, and this was not found. However, if the spatial Stroop effect is caused by temporal overlap between response codes associated with the target and response codes activated by the irrelevant location then the spatial Stroop effect should be magnified by alerting cues that increase this temporal overlap. As such, alerting cues may play a larger role when response codes associated with the irrelevant dimension fade rapidly and this should be seen in the data at earlier, relative to later, bins. A distributional analysis of the auditory modality found that the spatial Stroop effect increased across bins for RTs but decreased across bins for error rates. A similar pattern has been reported previously in a vertical version of the Simon task (Töbel et al., 2014) and may indicate that irrelevant response codes are activated early, as evidence by the fact that fast errors were strongly affected by the irrelevant dimension, but this information does not fade rapidly (or get inhibited), since slower RTs continued to be affected by the irrelevant dimension. However, in the visual modality, the spatial Stroop effect decreased across bins in both RTs and error rates, which supports the position that visual codes decay more quickly. Finally, a comparison of the error rate data across the two modalities supports the hypothesis that the alerting × spatial Stroop effect interaction decreases at later RTs with visual, but not auditory, stimuli (Fig. 5). As such we favor this explanation of the modality differences observed here. In addition, we note that this explanation is not reliant on the need for directional (e.g., left/right) associations. Although some studies report that directional associations are crucial (e.g., Kahan & Zhang, 2019; Schneider, 2019) there have been studies that report an alerting–congruency interaction in flanker tasks where stimulus–response associations exist from prior learning (participants respond with left and right button presses to unpleasant and pleasant words, respectively) but these associations are not clearly directional (Fischer et al., 2012). We note, however, that for right-handed participants negative words are associated with the left side of space and positive words are associated with the right (Casasanto, 2009). As such, valence decisions may be affected by pre-existing directional associations in the same way as the SNARC effect (spatial–numerical association of response codes) where many individuals associate smaller numbers with the left and larger numbers with the right (Kahan & Zhang, 2019). Irrespective of whether directional associations are necessary, or not, our data are consistent with the claim that alerting signals facilitate processing in early stages of vision and this ease of processing in turn facilitates the conversion of stimulus codes into associated response codes (Fischer et al., 2013).^12^ If the ease of extracting response codes is enhanced by the alerting cue and if response codes associated with the irrelevant dimension temporally overlap with those of the target then an alerting × congruency interaction ought to be found.

Other explanations for the alerting × congruency interaction have been offered but none of these can explain the effects reported here. For example, it has also been proposed that alerting cues may impair cognitive control making it harder to ignore distractors (Callejas et al., 2004, 2005), but this account cannot explain why the alerting–congruency interaction depends on stimulus–response motor associations (Fischer et al., 2012; Kahan & Zhang, 2019), nor can this explain the modality effects reported here. Finally, another possibility is that alerting cues broaden spatial attention (Weinbach & Henik, 2011), or enhance spatial grouping (Schneider, 2018), and although alerting cues seem to have these effects in some experiments, these possibilities alone do not explain why the irrelevant dimension must engage stimulus–response associations nor do these explanations account for modality differences.

We hypothesize that the modality differences reported here result from differences in the speed that response codes associated with auditory and visual targets decay, and the extent to which these codes can be enhanced and extended by alerting signals. Our data make it clear that the alerting × spatial Stroop effect interaction differs across modalities and support the position that alerting cues facilitate the conversion of stimulus codes into response codes and delay the rate of decay of response codes associated with the irrelevant dimension that might otherwise decay quickly.

## Footnotes

1 Explanations of the alerting-congruency interaction are reviewed, and evaluated, in the General Discussion.

2 There are a few notable counterexamples however where an alerting-congruency interaction has been found with stimuli that do not have pre-existing directional associations. These findings are discussed in relation to the current results in the General Discussion.

3 Böckler et al. (2011) had participants respond to visual shapes (squares or diamonds), and Soutschek et al. (2013) had participants respond to visual letters (‘‘X’’ or ‘‘O’’).

4 Demographic information of the sample in Experiments 1 and 2 were not recorded but the demographics of the population from which these samples were drawn are such that over 95% of students at the College are between the ages of 18 and 22 with a mean age of 20.2.

5 Schneider (2020) used a sample sizes of 60 and Fischer et al. (2010) used a sample size of 26 in studies examining the alerting-congruency interaction in visual versions of the spatial Stroop task. Similarly, in studies examining the alerting-congruency interaction in visual versions of the Simon task, Böckler et al. (2011) had a sample size of 16, and Soutschek et al. (2013) had a sample size of 26.

6 We recognize that with each additional analysis the overall Type 1 error rate increases. Nonetheless, we believe these distributional analyses, which show a pattern of results that are consistent with prior studies, improve our understanding of the effects reported in this experiment as well as Experiment 2.

7 For completeness, we report all of the non-significant interactions from this 2 × 2 × 5 repeated measures ANOVA of the RT data here. The interaction between alerting and spatial Stroop condition was *F*(1,97) = 0.49, *p* = .486. The interaction between alerting and bin was *F*(4,388) = 0.81, *p* = 0.520. The interaction between spatial Stroop condition and bin was *F*(4,388) = 1.25, *p* = 0.291. The three-way interaction between alerting, spatial Stroop condition, and bin was *F*(4,388) = 0.25, *p* = 0.910.

8 The non-significant interactions from this ANOVA are reported here. The interaction between alerting and spatial Stroop condition was *F*(1,97) = 1.36, *p* = 0.246. The interaction between alerting and bin was *F*(3,291) = 0.98, *p* = 0.410. The three-way interaction between alerting, spatial Stroop condition, and bin was *F*(3,291) = 0.81, *p* = 0.491.

9 This analysis yielded two additional non-significant effects, which are reported here for completeness. The main effect of alerting was *F*(1,97) = 0.57, *p* = 0.451. The interaction between alerting and spatial Stroop condition was *F*(1,97) = 1.07, *p* = 0.303.

10 We report all of the non-significant interactions from this ANOVA here. The interaction between alerting and spatial Stroop condition was *F*(1,95) = 0.25, *p* = 0.616. The interaction between alerting and bin was *F*(4,380) = 0.18, *p* = 0.950. The interaction between spatial Stroop condition and bin was *F*(4,380) = 0.66, *p* = 0.623. The three-way interaction between alerting, spatial Stroop condition, and bin was *F*(4,380) = 0.34, *p* = 0.850.

11 The non-significant interactions from this 2 × 2 × 4 ANOVA are reported here. The interaction between alerting and bin was *F*(3, 285) = 1.40, *p* = 0.243. The three-way interaction between alerting, spatial Stroop condition, and bin was *F*(3, 285) = 0.25, *p* = 0.859.

12 We thank Rico Fischer for bringing this possibility to our attention.
